# Supplementary material for: Double burden of malnutrition and associated factors among adolescent in Ethiopia: A systematic review and meta-analysis
Source: PLoS One. 2023 Apr 12;18(4):e0282240. doi: 10.1371/journal.pone.0282240 (PMC10096189; doi:10.1371/journal.pone.0282240)
Supplement: S1 Table — (DOCX) [file pone.0282240.s002.docx]

S1 Table. : JBI Critical Appraisal Checklist for Studies included in systematic review and meta-analysis of adolescent malnutrition in Ethiopia

| **Descriptive cross sectional studies** | | **Articles** | | | | | | | | | | | | | | | | | | | | | | | | | | | | | | |  |
| --- | --- | --- | --- | --- | --- | --- | --- | --- | --- | --- | --- | --- | --- | --- | --- | --- | --- | --- | --- | --- | --- | --- | --- | --- | --- | --- | --- | --- | --- | --- | --- | --- | --- |
|  | JBI Checklists | Abate B, et al | Alemu E, et al | Arage G. et al | Assefa et al | Berbada, D et al | Berhe, K.et al | Damie, T et al | Demilew, Y et al | Engidaw, M. et al | Gali, N.et al | Gebregyorgis, T.el | | Gebremariam, H et al | Hassen, K. etal | Irenso, A.et al | Jikamo, B. et al | Kahssay, M. et al | Kt, Roba et al | melaku, Y. A.et al | Roba, A. C.et al | Tariku, A. et al | Teferi, D. et al | Tegegne, M, etal | Teshome, T et al | Wakayo, T et al | Wassie, M et al | Woday, A et al | Yebyo H et al | Yemaneh, Y et al | Yetubie, M.et al | Zemene, M et al | |
| 1 | Was the sample frame appropriate to address the target population? | 1 | 1 | 1 | 1 | 1 | 1 | 1 | 1 | 1 | 1 | 1 | | 1 | 1 | 1 | 1 | 1 | 1 | 1 | 1 | 1 | 1 | 1 | 1 | 1 | 1 | 1 | 1 | 1 | 1 | 1 | |
| 2 | Were study participants sampled in an appropriate way? | 1 | 1 | 0 | 1 | 1 | 1 | 1 | 1 | 1 | 1 | 0 | | 1 | 1 | 1 | 1 | 1 | 1 | 1 | 0 | 1 | 1 | 1 | 1 | 1 | 1 | 1 | 0 | 1 | 1 | 1 | |
| 3 | Was the sample size adequate? | 1 | 1 | 1 | 1 | 1 | 1 | 1 | 1 | 1 | 1 | 1 | | 1 | 1 | 1 | 1 | 1 | 1 | 1 | 1 | 1 | 1 | 1 | 1 | 1 | 1 | 1 | 1 | 1 | 1 | 1 | |
| 4 | Were the study subjects and the setting described in detail? | 1 | 1 | 1 | 1 | 0 | 1 | 1 | 1 | 1 | 1 | 1 | | 1 | 0 | 1 | 1 | 1 | 1 | 1 | 1 | 1 | 0 | 1 | 1 | 1 | 1 | 1 | 1 | 1 | 0 | 1 | |
| 5 | Was the data analysis conducted with sufficient coverage of the identified sample? | 1 | 1 | 1 | 1 | 1 | 1 | 1 | 1 | 1 | 1 | 10 | | 1 | 1 | 1 | 1 | 1 | 1 | 1 | 1 | 1 | 1 | 1 | 1 | 1 | 1 | 1 | 1 | 1 | 1 | 1 | |
| 6 | Were valid methods used for the identification of the condition? | 1 | 1 | 1 | 0 | 1 | 1 | 1 | 1 | 1 | 1 | 1 | | 0 | 1 | 1 | 1 | 1 | 1 | 1 | 1 | 0 | 1 | 1 | 1 | 1 | 1 | 1 | 1 | 0 | 1 | 1 | |
| 7 | Was the condition measured in a standard, reliable way for all participants? | 1 | 0 | 0 | 1 | 1 | 1 | 1 | 1 | 1 | 0 | 0 | | 1 | 1 | 1 | 1 | 1 | 1 | 0 | 0 | 1 | 1 | 1 | 1 | 1 | 1 | 0 | 0 | 1 | 1 | 1 | |
| 8 | Was there appropriate statistical analysis? | 1 | 1 | 1 | 1 | 1 | 1 | 1 | 1 | 1 | 1 | 1 | | 1 | 1 | 1 | 1 | 1 | 1 | 1 | 1 | 1 | 1 | 1 | 1 | 1 | 1 | 1 | 1 | 1 | 1 | 1 | |
| 9 | Was the response rate adequate, and if not, was the low response rate managed appropriately? | 1 | 0 | 1 | 1 | 0 | 1 | 0 | 1 | 1 | 0 | 1 | | 1 | 0 | 1 | 0 | 1 | 1 | 0 | 1 | 1 | 0 | 1 | 0 | 1 | 1 | 0 | 1 | 1 | 0 | 1 | |
| Total % | | 100 | 78 | 67 | 89 | 78 | 100 | 89 | 100 | 78 | 78 | 67 | | 89 | 78 | 100 | 89 | 100 | 78 | 78 | 67 | 89 | 78 | 100 | 89 | 100 | 78 | 89 | 67 | 78 | 89 | 67 | |
| **Analytic cross sectional studies** | | **Articles** | | | | | | | | | | | | | | | | | | | | | | | | | | | | | | | |
|  | JBI Checklists | Abdulkadir A et al | | | | | | | | | | | Gebreyohannes, Y et al | | | | | | | | | | | | | | | | | | | | |
| 1 | Were the criteria for inclusion in the sample clearly defined? | 1 | | | | | | | | | | | 1 | | | | | | | | | | | | | | | | | | | | |
| 2 | Were the study subjects and the setting described in detail? | 1 | | | | | | | | | | | 1 | | | | | | | | | | | | | | | | | | | | |
| 3 | Was the exposure measured in a valid and reliable way? | 1 | | | | | | | | | | | 1 | | | | | | | | | | | | | | | | | | | | |
| 4 | Were objective, standard criteria used for measurement of the condition? | 1 | | | | | | | | | | | 1 | | | | | | | | | | | | | | | | | | | | |
| 5 | Were confounding factors identified? | 1 | | | | | | | | | | | 1 | | | | | | | | | | | | | | | | | | | | |
| 6 | Were strategies to deal with confounding factors stated? | 0 | | | | | | | | | | | 1 | | | | | | | | | | | | | | | | | | | | |
| 7 | Were the outcomes measured in a valid and reliable way? | 1 | | | | | | | | | | | 1 | | | | | | | | | | | | | | | | | | | | |
| 8 | Was appropriate statistical analysis used? | 1 | | | | | | | | | | | 1 | | | | | | | | | | | | | | | | | | | | |
| Total % | | 78 | | | | | | | | | | | 100 | | | | | | | | | | | | | | | | | | | | |
| **Case control study** | | **Article** | | | | | | | | | | | | | | | | | | | | | | | | | | | | | | | |
|  | JBI Checklists | Geta, M et al | | | | | | | | | | | | | | | | | | | | | | | | | | | | | | | |
| 1 | Were the groups comparable other than the presence of disease in cases or the absence of disease in controls? | 1 | | | | | | | | | | | | | | | | | | | | | | | | | | | | | | | |
| 2 | Were cases and controls matched appropriately? | 0 | | | | | | | | | | | | | | | | | | | | | | | | | | | | | | | |
| 3 | Were the same criteria used for identification of cases and controls? | 1 | | | | | | | | | | | | | | | | | | | | | | | | | | | | | | | |
| 4 | Was exposure measured in a standard, valid and reliable way? | 0 | | | | | | | | | | | | | | | | | | | | | | | | | | | | | | | |
| 5 | Was exposure measured in the same way for cases and controls? | 1 | | | | | | | | | | | | | | | | | | | | | | | | | | | | | | | |
| 6 | Were confounding factors identified? | 0 | | | | | | | | | | | | | | | | | | | | | | | | | | | | | | | |
| 7 | Were strategies to deal with confounding factors stated? | 1 | | | | | | | | | | | | | | | | | | | | | | | | | | | | | | | |
| 8 | Were outcomes assessed in a standard, valid and reliable way for cases and controls? | 1 | | | | | | | | | | | | | | | | | | | | | | | | | | | | | | | |
| Total % | | 70 | | | | | | | | | | | | | | | | | | | | | | | | | | | | | | | |
