# Supplementary material for: Double burden of malnutrition and associated factors among adolescent in Ethiopia: A systematic review and meta-analysis
Source: PLoS One. 2023 Apr 12;18(4):e0282240. doi: 10.1371/journal.pone.0282240 (PMC10096189; doi:10.1371/journal.pone.0282240)
Supplement: Attachment — Submitted filename: comments.docx [file pone.0282240.s004.docx]

- There were not the tables 1 to 4 in the file and I couldn’t see the list of studies and their score of critical appraisals.
- One of the most important of restrictions of this study is that it was restricted to only ‘English language’ which can lead to language bias and unreal estimation. It is recommended to include all published studies of Ethiopia adolescents regardless of the language of the articles
- Other weakness of study is that it included all studies without time restriction, while the malnutrition is a time depended issue and in order to get a true picture of the current state of society, it is better to apply a time restriction or do a cumulative meta-analysis to observe the changes in the index over time.
- It is recommended to use the random-effects model even if the heterogeneity is low, so it is recommended to use the random model for overweight/ obesity in this study as well.
- Have studies whose reports are not based on Z- Score been deleted?
