# Supplementary material for: Double burden of malnutrition and associated factors among adolescent in Ethiopia: A systematic review and meta-analysis
Source: PLoS One. 2023 Apr 12;18(4):e0282240. doi: 10.1371/journal.pone.0282240 (PMC10096189; doi:10.1371/journal.pone.0282240)
Supplement: Attachment — Submitted filename: response to reviewers.docx [file pone.0282240.s005.docx]

**Response to reviewers letter**

We are submitting our revised manuscript entitled “Double Burden of Malnutrition and Associated Factors among Adolescent in Ethiopia: A Systematic Review and Meta Analysis” for publication on PLOS One Journal. We hereby declare that the manuscript has never been submitted to any other Journal for consideration. All authors have read the manuscript and do not have any conflict of interest. We have addressed the comments given by the editor point by point as follows:

1. We have correctly cited all the tables and figures. Each figure caption appear directly after the paragraph in which they are first cited
2. Language restriction is due to interpretation purpose and almost all studies in Ethiopia published in English language. So language bias is less likely.
3. The protocol of this study was assuming that malnutrition is time dependent and plan to do cumulative meta-analysis, but all the studies done in Ethiopia were recent and we conceder no change over those studies.

Best regards,

Aragaw Gezaw

 Corresponding Author

Email: [aragawgezaw@gmail.com](mailto:aragawgezaw@gmail.com)

Telephone: +251921519095
