# Supplementary material for: Double burden of malnutrition and associated factors among adolescent in Ethiopia: A systematic review and meta-analysis
Source: PLoS One. 2023 Apr 12;18(4):e0282240. doi: 10.1371/journal.pone.0282240 (PMC10096189; doi:10.1371/journal.pone.0282240)
Supplement: Attachment — Submitted filename: Response to reviewers.docx [file pone.0282240.s006.docx]

**Response to reviewers letter**

We are submitting our revised manuscript entitled “Double Burden of Malnutrition and Associated Factors among Adolescent in Ethiopia: A Systematic Review and Meta-Analysis” for publication on PLOS One Journal. We hereby declare that the manuscript has never been submitted to any other Journal for consideration. All authors have read the manuscript and do not have any conflict of interest. We have addressed the comments given by the editor point by point as follow:

|  | **Comment** | | | **Response** | | | |
| --- | --- | --- | --- | --- | --- | --- | --- |
| **Abstract** | | | |  | | | |
|  | The author’s state: “Although there were many individual studies in Ethiopia, their results are inconclusive…” It would be helpful for the authors to specify what the studies are about – e.g., “studies in Ethiopia about [specify here – e.g., nutritional status among adolescents]” in order to improve clarity. | | | Although there were many individual studies in Ethiopia about different form of malnutrition among adolescent, their results are inconclusive indicating the need for generating a pooled estimate of adolescent nutritional status and associated factors. | | | |
|  | The authors write, “The prevalence of overweight/obesity, stunting and thinness are higher in Ethiopian adolescents…” The authors could consider instead writing that the prevalence was “high,” since the authors do not compare the prevalence of these forms of malnutrition in Ethiopian adolescents to the prevalence in another population. | | | The prevalence of overweight/obesity, stunting and thinness are high in Ethiopian adolescents indicating the upcoming challenge of double burden of malnutrition. | | | |
| **Introduction** | | | |  | | | |
|  | It would strengthen the paper for the authors to define the double burden of malnutrition, given the many definitions in the literature. It would also be useful for the authors to explicitly state the level at which they are assessing the double burden of malnutrition, since the double burden can occur at the country, household, or individual levels. | Malnutrition is deviations from the optimum body needs (it can be undernutrition or overnutrition). It is mainly caused by unbalanced, inadequate, or excessive intake of nutrients. Undernutrition refers to insufficient intake of dietary energy and nutrients that fulfill the body’s demand for optimum function.[^1^](https://journals.sagepub.com/doi/10.1177/0379572120968978#bibr1-0379572120968978) Undernutrition manifests in the form of stunting or wasting/thinness. Stunting is a chronic form of undernutrition which is caused by inadequate nutrition over a long period that fails to attain optimum growth while wasting/thinness is an acute form of undernutrition that indicates a recent food shortage and/or infectious diseases that leads to rapid and severe weight loss. Overnutrition include overweight and obesity which is abnormal or excessive accumulation of fat that may result in health impairment.  To assess the nutrition status of adolescents, the WHO currently recommends using BMI-for-age and height-for-age. Thinness (low body-mass-index (BMI)-for-age (BAZ)) z-score is below minus 2 (‑2.0) standard deviations (SD) and stunting (low height-for-age (HAZ)) z-score is below minus 2 (‑2.0) standard deviations (SD). Overweight is (high Body Mass Index (BMI)-for-age)) greater than plus 1 standard deviation; and obesity is greater than plus 2 standard deviations above the WHO Growth Reference median. | | | | | |
|  | Suggest re-wording the last sentence in the first paragraph for clarity. I believe the authors are referring to the idea that a continuum of care from childhood through adolescence is needed. | | Although adolescents need a continuum care from childhood through adolescent, they are often ignored | | | | |
| **Methods** | | | | | | | |
|  | In the information sources and search strategy section, the authors state that “the reference lists of the retrieved studies were probed to collect articles that were not accessible through databases and search engines.” Were they accessible but just not picked up by the search strategy? | | | | The reference lists of the retrieved studies were probed through contact relevant experts and organization’s website to collect articles that were not accessible through databases and search engines. | | |
|  | In the study selection section, the authors seem to describe how level of agreement was assessed for the title and abstract screening. I suggest they also describe how agreement was achieved for the full-text review. | | | | After reaching good agreement, a full-text review was performed. When there was disagreement the review resolved through discussion. | | |
|  | In the study selection section, I suggest the authors describe how disagreements were resolved – e.g., through discussion. | | | | After reaching good agreement, a full-text review was performed. When there was disagreement the review resolved through discussion. | | |
|  | In the data collection process, the authors mention that a “pilot test was conducted for all forms of using a representative sample of the studies to be reviewed…” What does it mean for the sample to be representative? | | | | Pilot test was conducted for all data extraction forms using a representative sample of the studies to be reviewed and the form was corrected based on pilot test finding.  Sample to be representative means before conducting data extraction representative sample study were taken and pilot study was done | | |
|  | I suggest the authors revise the quality appraisal and risk of bias section for clarity – e.g., the authors write that “the source of discrepancy was investigated through making a thorough revision.” Did the authors mean that disagreements were resolved through discussion? | | | | For any scoring disagreements, which were happening between the assessors, the source of discrepancy was investigated through making a thorough revision and disagreements were resolved through discussion. | | |
| Results | | | | | | | |
|  | The first sentence states that there were a total of 1289 records, while the figure indicates that there were a total of 1269 records. Is this a typo? | | | | In the initial search, a total of 1289 records were found from different electronic search databases, search engine and other sources:   - Search engine and other source = 20 and database = 1269 - So, Total records =1289 | | |
|  | Figures 16, 17, and 18 are difficult to read. They may need to be re-formatted. | | | | Re-formatted | | |
| **Discussion & Conclusion** | | | | | | | |
|  | Is the prevalence of overweight and obesity among Ethiopian adolescents increasingly rapidly? If so, what do the authors think about generating a pooled prevalence estimate based on studies conducted between 2010 – 2020? | | | | | | Trend analysis needed |
|  | The authors could consider moving the first paragraph of the discussion section to the introduction, as it describes the problem of malnutrition. | | | | | | Possible but it is the summary of the discussion part |
|  | The authors compare their findings to results from other LMICs and high-income countries. The authors could consider also describing how the results compare to those from other countries in the region. | | | | | | We got similar studies in the compared region  If there was similar studies in other region we can compare |
|  | The authors write “The pooled estimates of overweight/obesity showed high increase” and then later refer to the “rising prevalence of overweight/obesity.” Since trends were not evaluated in this study, I suggest not using words that indicate an increasing trend in order to ensure that the findings support the conclusions. | | | | | | The results imply the need for giving more emphasis on design and implementation of preventive policies to reduce stunting and thinness and the high prevalence of overweight/obesity among adolescent in Ethiopia. |
| Overall | | | | | | | |
|  | Since micronutrient deficiencies and associated morbidity and mortality are a persisting challenge in Ethiopia, it may strengthen the paper for the authors to discuss micronutrient deficiencies (even if briefly) in the paper (e.g., in the discussion section, the authors could consider noting that there are other indicators of nutritional status that are important to measure and address). | | | | | We did not study micronutrient deficiency | |

Best regards,

Aragaw Gezaw

 Corresponding Author

Email: [aragawgezaw@gmail.com](mailto:aragawgezaw@gmail.com)

Telephone: +251921519095
